# Comparative Study of the Gel-Forming Ability of Type I Collagens Extracted from Different Organs and Fish Species

**DOI:** 10.3390/gels11070533

**Published:** 2025-07-09

**Authors:** Abdul Ghani, Mantaro Okada, Beini Sun, Xi Zhang, Ichiro Higuchi, Yasuaki Takagi

**Affiliations:** 1 Graduate School of Fisheries Sciences, Hokkaido University, 3-1-1 Minato-cho, Hakodate 041-8611, Hokkaido, Japan; abdul.ghani.w3@elms.hokudai.ac.jp (A.G.); munchmunch0101@gmail.com (M.O.); beinisun1109@gmail.com (B.S.); 2 Faculty of Fisheries Sciences, Hokkaido University, 3-1-1 Minato-cho, Hakodate 041-8611, Hokkaido, Japan; zhangxi@mail.hzau.edu.cn (X.Z.); ichiro.higuchi@fish.hokudai.ac.jp (I.H.); 3 College of Fisheries, Huazhong Agriculture University, Wuhan 430070, China

**Keywords:** freshwater-derived collagen, gel-forming ability, fibril formation, biomedical applications

## Abstract

The gel-forming ability of collagens is vital for their application in cell scaffolds, yet very few comparative studies on fish collagen sources are available. This study isolated and characterized type I collagens from carp skin (CSK), scales (CSC), and swim bladders (CSB) and sturgeon skin (SSK) and swim bladders (SSB). The carp collagens exhibited higher thermal stability (34.75–34.78 °C) and formed more transparent, stronger gels than the sturgeon collagens. Additionally, as demonstrated by scanning electron microscopy, the sturgeon collagens exhibited faster fibril formation, with visible fibrils after 3 h which grew thicker but did not form bundles. The carp collagens, in contrast, initially displayed fewer, thinner, and longer fibrils, with their formation accelerating over time and fibril bundles emerging after 24 h. All collagen solutions of 4% (*w*/*v*) exhibited shear-thinning flow behavior, with the carp-derived solutions showing higher viscosities (10^3^–10^4^ Pa·s) than those demonstrated by the sturgeon-derived solutions (10^2^–10^3^ Pa·s). The CSBs and SSBs demonstrated the highest storage (G′) and loss (G″) moduli, with the former exhibiting the lowest loss tangent (tan δ), indicative of a stronger gel structure. The gels at 24 h showed slightly poorer mechanical properties than those at 3 h. The CSC and SSB gels had the highest thermal stability. These findings highlight the distinctiveness of the characteristics of collagens and their gels, emphasizing their potential in biomaterial applications. The present study also provides a foundational framework for assessing cellular responses in a comparative context that may help in identifying the most suitable collagen types for biomedical applications.

## 1. Introduction

Interest in biodegradable material is growing. Collagen-based hydrogels, mainly derived from land animals, like cows, pigs, and sheep, are widely used in medical fields, such as tissue engineering and wound dressings. However, concerns about religious restrictions and disease transmission, e.g., foot and mouth disease, transmissible spongiform encephalopathy, and bovine spongiform encephalopathy (BSE), have limited their use [1,2]. Consequently, the demand for alternative collagen sources, particularly from aquatic animals, is increasing. Marine- and freshwater-derived collagens are gaining interest from researchers because of their sustainability, safety, and broader cultural acceptance, making them promising alternatives for various applications [3].

By-products of aquaculture fish are gaining increasing attention as stable collagen sources due to the growing aquaculture industry. For example, the aquaculture of caviar-producing sturgeon is experiencing rapid growth, resulting in a notable increase in its global production in recent years [4,5]. Similarly, the common carp (*Cyprinus carpio*), the fourth most cultured freshwater fish globally, produces 4.24 million metric tons of by-products annually, accounting for 8.6% of the total global inland aquaculture production [6]. These industries generate substantial by-products that sometimes constitute 30–70% of the whole fish, depending on the species and type of processing [7]. Fish by-products are primarily treated as waste, which presents substantial disposal challenges. However, these by-products are a valuable source of high-market-value biomolecules, including collagen. Extracting such high-value biomolecules from aquaculture by-products also aligns with United Nations Sustainable Development Goal (SDG) 12, which encourages resource efficiency and waste reduction to minimize environmental impacts by emphasizing sustainable consumption and production patterns [8].

The remarkable diversity in type I collagens obtained from fish and their various by-products distinguishes them from their mammalian counterparts. This diversity arises from species-specific variations in the amino acid sequences of type I collagen α chains, leading to collagens with different properties that may prove highly useful for various medical applications. Moreover, unlike mammalian type I collagen, which is composed of a combination of two α1 chains and one α2 chain ((α1)_2_α2), fish collagen often includes a third α chain (α3) which allows multiple molecular combinations such as (α1)_2_α2, α1α2α3, α3_2_α2, and α3(I)_3_ [9]. Even within the same fish species, the type I collagens extracted from different organs show variations in their molecular compositions, which contribute to the distinct physicochemical properties of each collagen, influencing its potential applications [9]. Understanding this diversity is crucial for optimizing the utilization of fish collagen in various biomedical and industrial applications.

Numerous studies have documented the physicochemical and biochemical characteristics of collagens extracted from various organs of marine and freshwater fishes [10,11,12,13,14,15]. However, no study has yet characterized the comparative gel-forming ability of collagens from different fish species as well as from different organs of the same species. Gel-forming ability is a critical factor that determines the suitability of collagen for applications such as cell scaffold hydrogels, as mechanical strength, stability, and biocompatibility are essential for successful tissue engineering applications. The present study aimed to address this research gap by extracting collagen from the various by-products of carp (skin, scales, and swim bladders) and bester sturgeon (skin and swim bladders) and by characterizing their physicochemical properties, gel-forming abilities, and gel properties. By concentrating on these underutilized by-products, this study also aimed to advance the sustainable development of the fisheries and aquaculture sectors by promoting waste valorization. Furthermore, it endeavored to underscore the potential of collagen derived from fish waste as a viable biomaterial for applications in tissue engineering.

## 2. Results and Discussion

### 2.1. Amino Acid Compositions of the Collagens

The amino acid compositions of the extracted collagens are listed in Table 1. Amino acid composition influences the structural properties of collagen. For instance, glycine stabilizes the collagen triple helix by enabling the close packing of α-chains into a superhelix [16]. The imino acid (proline and hydroxyproline) contents are vital for maintaining the structural integrity of collagen. Proline contributes to the primary structure of peptides and helps in maintaining the tertiary structure of collagen. Hydroxyproline plays a major role in forming hydrogen bonds via hydroxyl groups [17,18].

The glycine contents in the sturgeon collagens were greater than those in the carp collagens, although the variations in the glycine levels of the organs within each species were small. In contrast, the contents of imino acids were relatively greater in the carp collagens than they were in the sturgeon collagens. Notably, the imino acid contents of the collagens derived from two different sturgeon organs were also different, whereas no substantial variation was noted among the carp collagens. The carp collagens had similar degrees of proline hydroxylation, at approximately 40%. In contrast, the sturgeon collagens had different degrees of proline hydroxylation (34% in the SSK collagen and 45% in the SSB collagen). Thus, the lower imino acid contents and the lowest degree of proline hydroxylation in the SSK collagen resulted in the lowest hydroxyproline contents. In contrast, the highest proline hydroxylation rate led to the greatest hydroxyproline contents in the SSB collagen. Thus, both the organ-specific numbers of imino acid residues, which are led by the organ-specific α chain compositions, and the organ-specific degrees of proline hydroxylation defined the organ-specific hydroxyproline contents in the same species. The present study also confirmed the importance of hydroxyproline residues on the organ-specific thermal stability of collagen molecules and gels (Section 2.2 and Section 2.7), confirming the suggestion made by Zhang et al. [9].

### 2.2. Thermal Stability of Collagen Molecules

**Figure 1:**
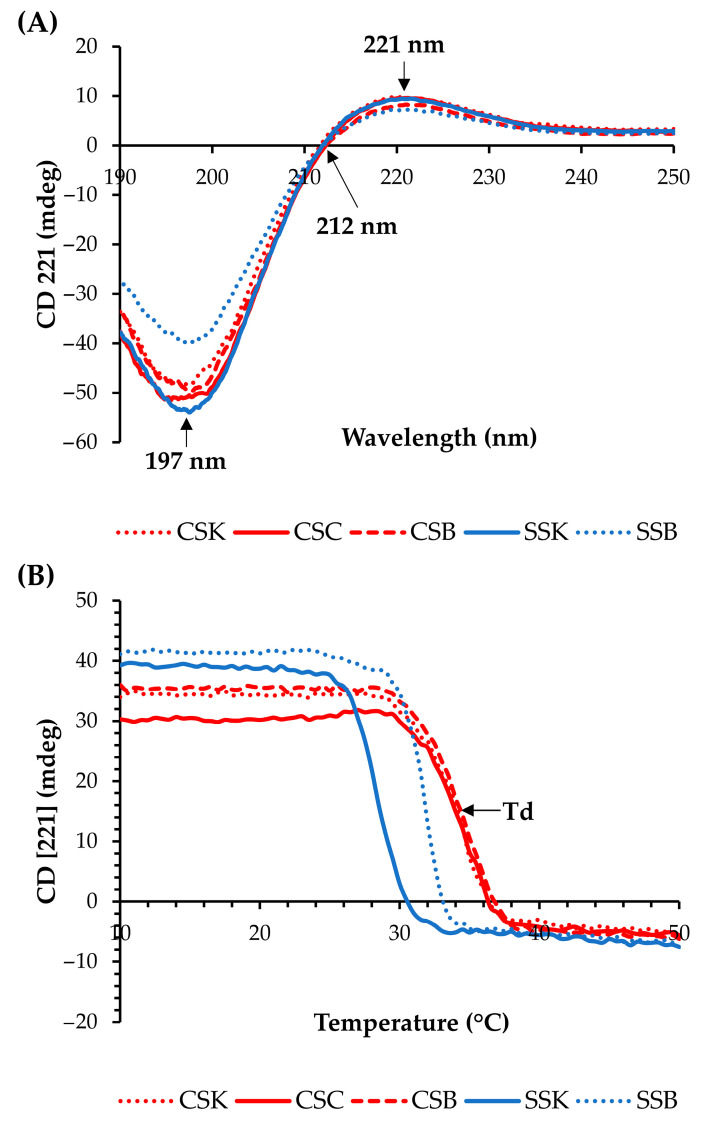
CD spectra (**A**) and temperature effect on the CD spectra at 221 nm (**B**) of the collagens from carp skin (CSK), scales (CSC), and swim bladders (CSB) and sturgeon skin (SSK) and swim bladders (SSB).

Circular dichroism (CD) spectra were used to confirm the secondary structures of the collagens (Figure 1A). All collagens showed positive peaks at approximately 221 nm, zero rotations (crossover point) at 212 nm, and minimum negative peaks at approximately 197 nm, indicating that the collagen molecules exhibited triple helical conformations [4,19,20].

The molar ellipticity at 221 nm decreased as the temperature increased due to the breakdown of the triple helical structure (Figure 1B). The denaturation temperature (Td) was determined as the temperature with the fastest decrease in the CD (221) value [4]. The carp collagens showed higher Tds than the sturgeon collagens, and the Tds of the CSK, CSC, CSB, SSK, and SSB collagens were 34.75, 34.75, 34.78, 28.25, and 31.75 °C, respectively. Thus, the organ differences in the Tds within the same species were large in the sturgeon but minor in the carp. Many studies have reported Tds of <30 °C for fish collagens [4,12,21]. In contrast, our study demonstrated comparatively high denaturation temperature values (>30 °C) of the CSK, CSC, CSB, and SSB collagens, which proved their relatively good structural stability against heat. Hence, these collagens can be considered as highly effective alternatives to mammalian collagens for biomaterial applications.

### 2.3. Viscosity of the Collagen Solutions

**Figure 2:**
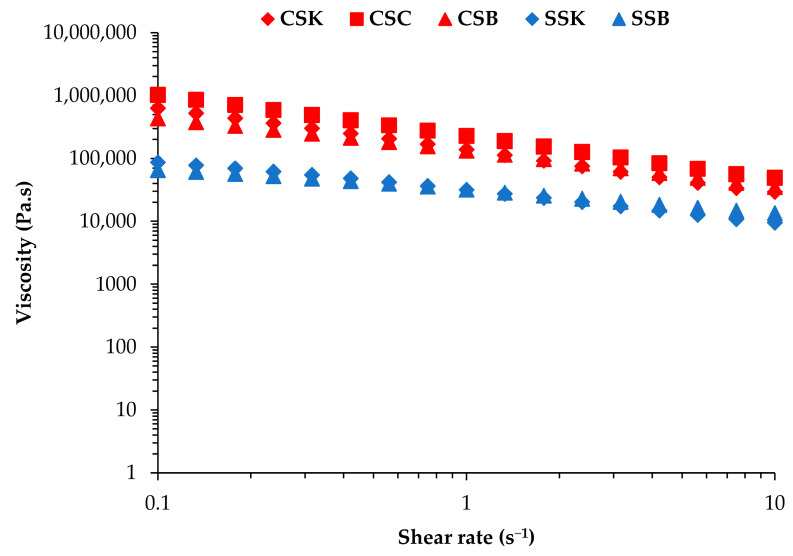
The dynamic viscosity (at 20 °C) of the acidic solutions of the collagens from carp skin (CSK), scales (CSC), and swim bladders (CSB) and sturgeon skin (SSK) and swim bladders (SSB), with the shear rate ranging from 0.1 to 10 s^−1^.

The viscosity profiles of the collagen solutions were evaluated as a function of their shear rate at a fixed concentration of 4% (*w*/*v*) (Figure 2). All solutions exhibited shear-thinning flow behavior, with viscosity decreasing almost linearly with increasing shear rate, similar to trends reported in previous studies on collagen solutions [22,23]. The viscosities of the carp collagens were much higher than those of the sturgeon collagens, and the CSC collagen exhibited the highest viscosity, followed by the CSB and CSK collagens. In contrast, the viscosity of the SSK collagen was higher than that of the SSB collagen. Therefore, the species and organ differences were apparent. However, the species differences were more prominent, and the organ differences were species-specific. The higher viscosities of the carp collagen solutions could be attributed to the more extensive molecular entanglements in the acidic solvent, although the factors contributing to such species differences are not known. Previous studies have reported exponential increases in collagen viscosity with concentration, particularly above critical thresholds such as 1.5% (*w*/*w*) in bovine hide type I collagen [23] and 2% (*w*/*w*) in bovine and porcine skin type I collagen [22]. Thus, the concentration dependency of the viscosities in our samples could help clarify the organ-specific relationships between the viscosity and the collagen concentrations.

### 2.4. Fabrication of the Collagen Gels

**Figure 3:**
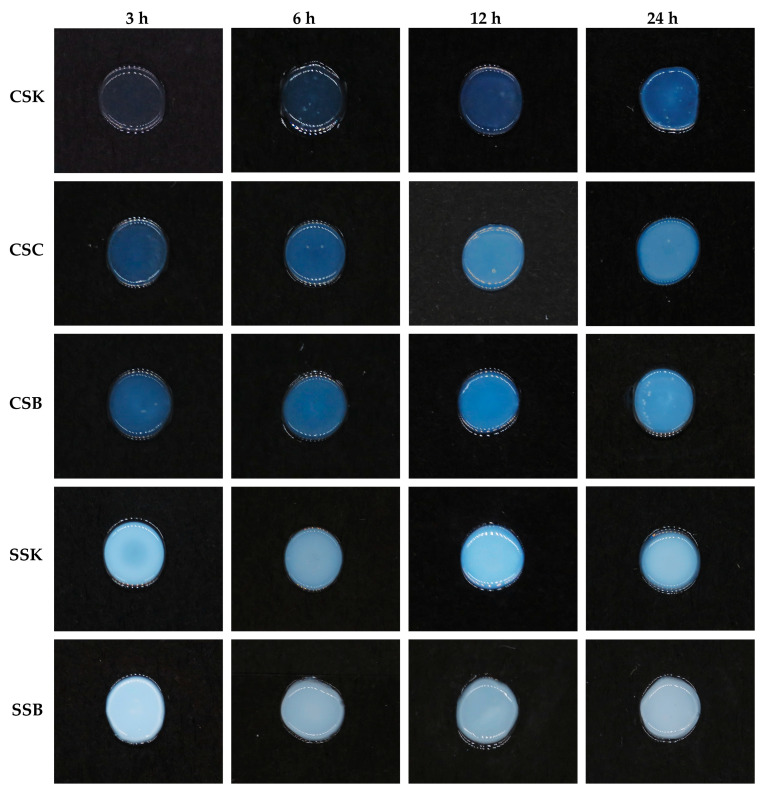
Photographic images of the 4% (*w*/*v*) hydrogels made of the collagens obtained from carp skin (CSK), scales (CSC), and swim bladders (CSB) and sturgeon skin (SSK) and swim bladders (SSB) at different gelation timepoints (3, 6, 12, and 24 h) at 20 °C.

All collagen solutions initiated immediate gelation at the periphery upon contact with 0.1 M of PB (pH of 7.2). The gelation advanced inward from the outer edge as the buffer gradually diffused into the collagen solution, eventually forming a disk-shaped gel after at least 3 h (Figure 3). The gels exhibited significant differences in their appearances and stabilities among the fish species and within the organs within each species. The carp gels were more transparent and demonstrated better stability than the sturgeon gels at the same timepoint. However, the transparency of the carp gels decreased with increasing gelation time, which may have indicated progressive fibril formation. In contrast, no such changes in transparency were observed in the sturgeon gels; they were opaque at the earliest timepoint tested in this experiment (3 h), suggesting that fibril formation was likely complete after 3 h of gelation. In contrast to a previous study that focused on external factors influencing gelation in bester sturgeon swim bladder collagen [22], the current study revealed for the first time that intrinsic differences in the collagen sources, particularly between the carp and sturgeon, as well as among their organs, significantly affected gel transparency, stability, and structural integrity.

### 2.5. Collagen Gel Microstructure

**Figure 4:**
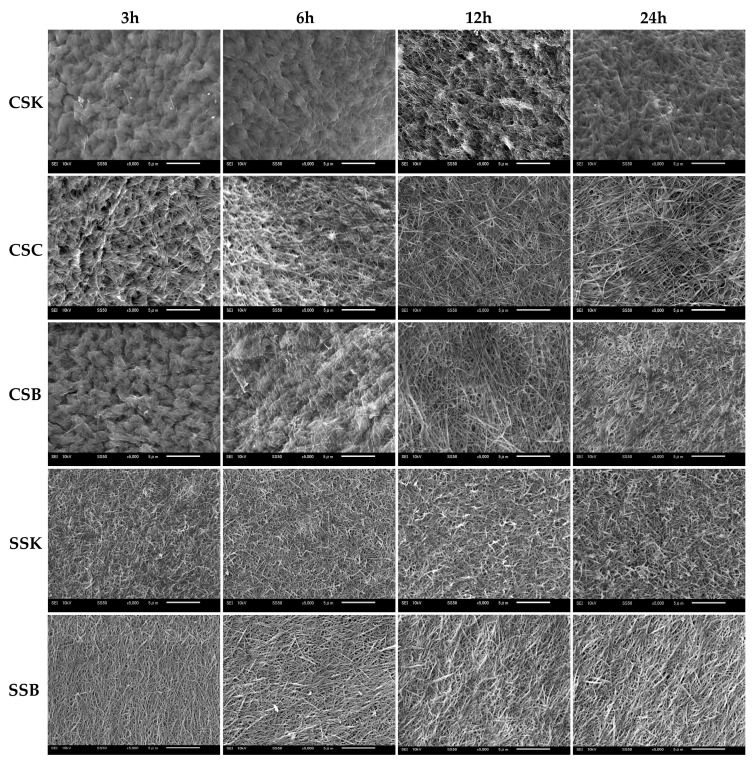
Surface SEM images of the 4% (*w*/*v*) hydrogels made of the collagens derived from carp skin (CSK), scales (CSC), and swim bladders (CSB) and sturgeon skin (SSK) and swim bladders (SSB) at different gelation timepoints (3, 6, 12, and 24 h) at 20 °C. The scale bars = 5 µm.

Figure 4 illustrates the microstructures of the gels at four distinct gelation timepoints (3, 6, 12, and 24 h). The fibril microstructure is an essential factor that determines the biomedical properties of a hydrogel, as the surface microarchitecture of collagenous cell scaffolds plays a key role in regulating cellular behavior [24,25].

The sturgeon gels showed faster fibril formation than the carp gels, with well-defined fibrils observed at 3 h of gelation. Over time, the fibrils became thicker and shorter. In the SSK collagen, the fibrils were short, thin, and randomly oriented, with no significant changes over time. In contrast, the SSB collagen fibrils were slightly longer, thicker, and more aligned. In addition, their thickness further increased with prolonged gelation, which was consistent with previous findings [9,22]. In contrast, the carp collagens initially displayed fewer fibrils and rougher surface microstructures at 3 h of gelation, indicating that fibril formation had started but could not be completed. However, the fibril formation accelerated with time, and by 24 h, all carp-derived gels exhibited fibril bundles, likely formed by fibril fusion. Before the bundle formation, the carp fibrils were thinner, longer, and more randomly oriented than those in the sturgeon gels. Regarding the carp organs, the CSC collagen exhibited the fastest fibril formation, followed by the CSB and CSK collagens, with almost similar fibril morphologies in length, orientation, and thickness.

These observations suggest that the speed, alignment, and morphology of fibril formation in fish-derived collagen is influenced by species, organ, and gelation time. Furthermore, the data highlight that gel formation and fibrillogenesis are distinct processes, and the completion of gel formation does not necessarily indicate the completion of fibril formation.

### 2.6. Mechanical Properties of the Collagen Gels

**Figure 5:**
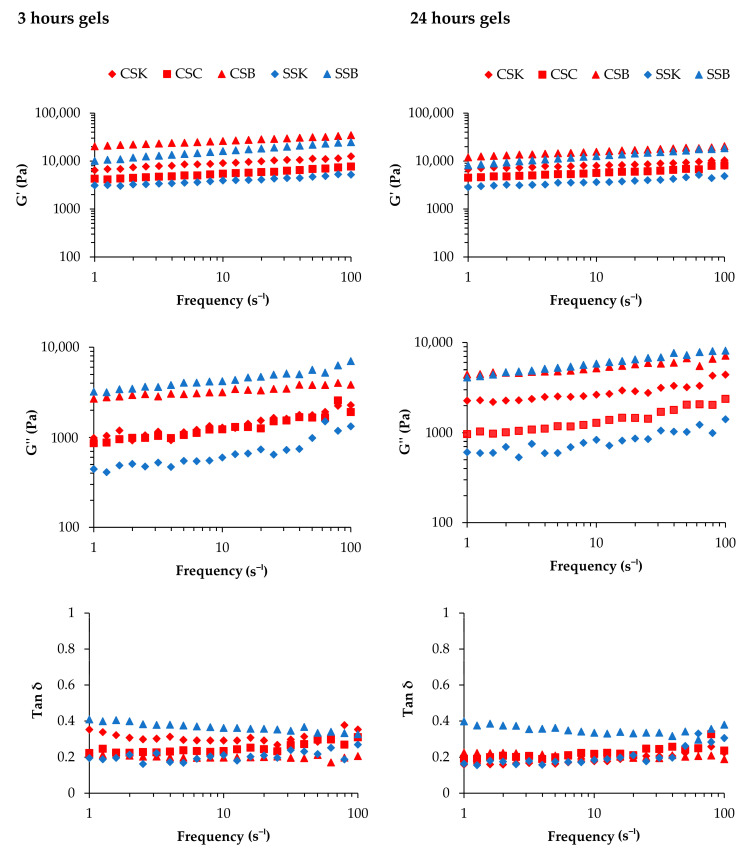
Storage modulus (G′), loss modulus (G″), and loss tangent (tan δ) of the 4% (*w*/*v*) hydrogels made of collagens derived from carp skin (CSK), scales (CSC), and swim bladders (CSB) and sturgeon skin (SSK) and swim bladders (SSB) at 3 and 24 h gelation timepoints at 20 °C.

The mechanical strength of a hydrogel is a crucial factor that determines its suitability for tissue engineering applications [26]. Figure 5 illustrates the storage modulus (G′), loss modulus (G″), and loss tangent (tan δ) as a function of frequency at two different gelation times (3 and 24 h).

The G″ values for all gel types were significantly lower than their corresponding G′ values in both the 3 h and 24 h gelation samples, indicating the viscoelastic nature of the gels. These findings were consistent with those of previous studies on SSB gels at 4 wt% [22] and calf skin collagen/oxidized chondroitin sulfate (CSox) hydrogels with 0, 0.125, 0.250, 0.500, and 2.000 mg/mL of CSox at a collagen concentration of 5 mg/mL [27].

When the gelation time was 3 h, the CSB and SSB gels exhibited the highest G′ values in the same species across all frequencies, indicating the superiority of swim-bladder gels in elasticity compared to the gels derived from skin and scales. Thus, the organs from which collagen is extracted affect the G′ value. The species difference was also evident, as the carp gels showed higher G′ values within the same organ. Similarly, the CSB and SSB gels exhibited the highest G″ values in the same species, reflecting greater energy dissipation during deformation than the skin and scale gels. The G″ values for both the CSC and CSK gels were higher than the SSK gel but lower than those of the CSB and SSB gels. Despite the SSB gel having a high G′ value, its elevated tan δ indicated the relative weakness of the gel compared to those of the other gels. Hence, it could be suggested that the SSB gel was elastic but lacked mechanical strength.

The species and organ trends regarding the G′, G″, and tan δ values of the gels obtained after 24 h of gelation were similar to those of the 3 h gelation samples. Notably, the general trends regarding the reduction in G′ and G″ values were comparable to those of the 3 h gelation samples. An apparent decrease in the G″ value of the CSK gel with a large decrease in tan δ was observed. This decrease might happen over time when collagen molecules self-assemble into tighter fibril bundles, resulting in a denser and more compact gel. This process reduces viscous dissipation (G″), making the gel more elastic and solid-like (lower tan δ). In addition, as the collagen network tightens, it may expel bound water, further increasing the gel’s stiffness and potentially causing a significant drop in G″ and tan δ values.

In summary, significant differences in the G′, G″, and tan δ values were observed between the species and among the organ types, with greater variations in the sturgeon-derived gels. The reduced mechanical properties with extended gelation times may be attributed to the phase transition of the collagen molecules into fibrils and an increase in the thickness of the fibrils. As the fibril thickness increased, the number of fibrils must have decreased because the lateral growth of the fibrils may have occurred by the lateral fusion of each fibril. Such reductions in the number of fibrils would decrease the interactions and attachments among the fibrils, resulting in a weaker and less rigid gel network.

### 2.7. Gel Denaturation Temperature

**Figure 6:**
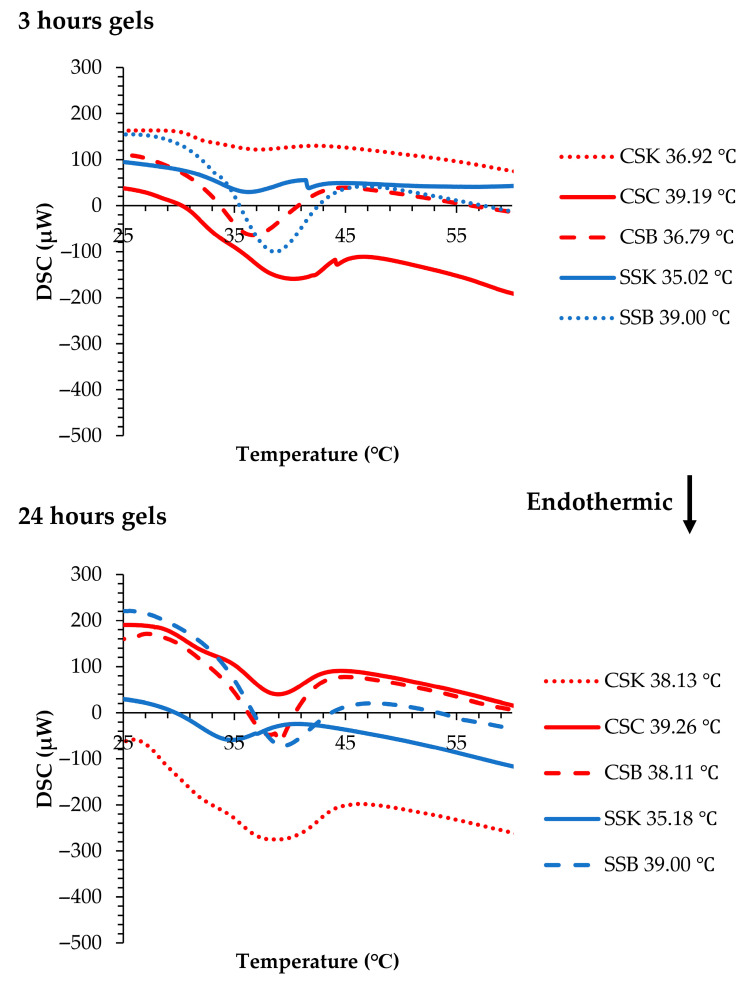
Differential scanning calorimetry curves of the 4% (*w*/*v*) hydrogels made of collagens obtained from carp skin (CSK), scales (CSC), and swim bladders (CSB) and sturgeon skin (SSK) and swim bladders (SSB) at 3 and 24 h gelation timepoints at 20 °C.

The thermal stability of collagen gel is a critical factor in biomedical applications, such as tissue engineering, where prolonged mechanical integrity is required [28]. Figure 6 illustrates the Tds of the gels. In the carp gels, after 3 h of gelation, the highest Td was observed in the CSC gel (39.19 °C), followed by CSK (36.92 °C) and CSB (36.79 °C) gels. In the sturgeon gels, the SSB gel showed a higher Td (38.53 °C) than the SSK gel (35.02 °C).

When compared with the corresponding collagen molecules measured via CD spectroscopy (CSC, 34.75 °C; CSK, 34.75 °C; CSB, 34.78 °C; SSK, 28.25 °C; and SSB, 31.75 °C) (Figure 1B), the increase in Td after gelation was substantial. This shift confirmed that gelation contributes to structural stabilization, also has been reported in previous studies on fish-derived collagen hydrogels [22]. Previous work [22] using sturgeon swim bladder collagen (SBC) and standard samples such as calf skin collagen (CSC) and bovine skin collagen (BSC) reported a similar pattern, where the Td of the SBC hydrogel increased dramatically from 32.9 °C (CD) to 43 °C (DSC), indicating the formation of thick, stable fibrils via diffusion-induced gelation. In contrast, the calf and bovine skin collagens showed smaller Td shifts (37 to 41.5 °C and 41.3 to 43 °C, respectively), suggesting less-efficient fibril network formation [22].

In our samples, the rate of Td increase exhibited both species and organ specificity. The sturgeon gels showed higher rates of increase, with minor organ differences, whereas the carp gels showed relatively lower increases, except for the CSC gel, which demonstrated a notably higher rate. These trends suggest that the degree of Td elevation corresponds to the extent of fibril formation because the sturgeon collagens showed a higher degree of fibril formation after 3 h of gelation. The CSC collagen also showed relatively faster fibril formation compared to the CSK and CSB collagens (Figure 4). Notably, the gels with higher Tds, such as the SSB and CSC gels, also exhibited higher G′ values (Figure 5), indicating a stiffer, more elastic network. However, despite the high G′ value of the SSB gel, its elevated tan δ value (Figure 5) revealed lower mechanical strength, indicating that while the gel was elastic, it may also have been structurally less rigid or cohesive than the others.

After 24 h of gelation, the Tds further increased compared to the carp gels after 3 h. The Td of the CSB gel increased from 36.79 to 38.11 °C, while that of the CSK gel rose from 36.92 to 38.13 °C. The CSC gel showed only a minor increase, from 39.19 to 39.26 °C. In contrast, the sturgeon gels exhibited minimal changes in Tds, supporting the hypothesis that the sturgeon collagen completed most of its fibril formation within 3 h. Afterward, only lateral fibril fusion contributed to network maturation. This interpretation aligned with mechanical data showing reduced G″ and tan δ values in some gels (e.g., the CSK gels), reflecting tighter fibril packing and reduced viscous dissipation, which resulted in more solid-like behavior.

The enthalpy (ΔH) after 3 h of gelation was the highest for the SSB and CSC gels, exhibiting 2.30 J/g and 1.88 J/g, respectively, indicating a greater degree of structural organization and more extensive intermolecular bonding within the fibril network in these gels. In contrast, the CSK and SSK gels showed lower ΔH values, 0.27 J/g and 0.39 J/g, respectively, suggesting less developed gel structures. Upon extending the gelation time to 24 h, the ΔH values increased in most samples, particularly in the CSK gel (from 0.27 to 2.35 J/g) and CSB gel (from 0.84 to 1.18 J/g), reflecting ongoing fibril formation and enhanced network consolidation over time. The relatively minor change in the CSC (1.88 to 1.89 J/g) and SSB (2.30 to 2.32 J/g) gels supported the earlier observation that these gels reached near-complete structural development within the initial 3 h. These enthalpy trends aligned with the corresponding Td shifts, reinforcing the conclusion that the thermal behavior of collagen gel is strongly influenced by the extent and kinetics of the fibril formation. DSC-derived enthalpy has similarly been used to evaluate structural stability in other polymer systems. In a study on a cambium gum-based encapsulation matrix, a DSC analysis was used to assess thermal degradation behavior, demonstrating that the energy required to disrupt the material’s structure reflected the degree of internal cohesion [29].

Overall, the carp collagen gels showed higher thermal stability with less variation among the organs, whereas the sturgeon collagens showed higher variability among the different organs. These differences emphasized the species- and organ-specific variations in the thermal behaviors of the collagen gels.

## 3. Conclusions

Carp and sturgeon collagens exhibit distinct gelation behaviors, primarily influenced by species-specific characteristics rather than organ-specific differences. Generally, carp-derived gels demonstrate higher stability, transparency, mechanical strength, and denaturation temperature than those derived from sturgeon. In addition, carp-derived gels form fibrils more slowly, with thin, long, randomly oriented structures that eventually bundle over time. In contrast, sturgeon-derived gels form fibrils more rapidly, and these remain thicker and shorter. Apart from species variations, organ-specific differences also influence the properties of collagen gels. In sturgeon, fibril morphology varies by organ type, whereas in carp, fibril formation speed is organ-specific. Therefore, fish collagen can produce a wide range of gels with different characteristics that can be used in biomedical research and applications. Additionally, by promoting the use of fishery and aquaculture by-products, this study highlights fish collagen as a sustainable and promising biomaterial source for biomedical applications. The present study employed a pepsin-aided HCl extraction method for collagen; however, different collagen extraction methods (pepsin-aided acetic acid extraction, deep eutectic solvent extraction, supercritical fluid extraction, extrusion and ultrasound-assisted extraction, etc.) that result in varying yields have been reported. Comparative studies on the gel properties of collagens extracted by different methods remain a future task.

## 4. Materials and Methods

Unless otherwise specified, the standard procedures in this section were followed with slight modifications as described in previous studies [4,9,22,30].

### 4.1. Raw Materials and Sample Preparation

Four live cultured carp fish (*Cyprinus carpio*) averaging 1.40 ± 0.18 kg in weight and 42.17 ± 1.66 cm in length were procured from Miyazaki Fish Shop located at Onuma Lake, Nanae, Hokkaido, Japan, and transported to the laboratory under aerated conditions. Before organ processing, all fish underwent deep anesthesia induced by immersion in a 2-phenoxyethanol solution (0.5 mL/L). Subsequently, the scales were removed from each fish using tweezers, and the skin was excised with a knife. Their swim bladders were carefully dissected. Each organ type from all four fish was combined, rinsed with cold distilled water (4 °C), divided into equal portions, placed in polyethylene bags, and stored at −80 °C until collagen extraction. The bester sturgeon (*Huso huso* × *Acipenser ruthenus*), a hybrid species of sturgeon cultured in Bifuka Town, Hokkaido, Japan, was also used. Fresh skin and swim bladder samples were collected from a food workshop in Bifuka. These samples were subsequently packed, frozen, and transported to the Faculty of Fisheries Sciences, Hokkaido University, Hokkaido, Japan. The samples were stored at −80 °C until collagen extraction.

### 4.2. Extraction and Purification of the Collagen

All procedures were conducted at 4 °C or at ice-cold conditions. Firstly, the frozen samples were thawed overnight, washed with cold distilled water, and cut into approximately 0.5 × 0.5 cm pieces. The skin and swim bladder samples were stirred for 24 h in 0.1 M NaOH with a sample/solution ratio of 1:50 (*w*/*v*). The carp scales were similarly treated but with a sample/solution ratio of 1:30. The alkali solution was changed every 8 h. Then, the alkali-treated skin was defatted in 99.5% ethanol at a sample/solution ratio of 1:25 (*w*/*v*) at 4 °C with continuous stirring for 48 h. The ethanol solution was changed every 12 h. The scales underwent decalcification using 0.5 M 2Na-EDTA (pH pf 7.4) at a sample/solution ratio of 1:20 (*w*/*v*) for 48 h, and the solution was changed every 12 h. After each treatment, the specimens were washed repeatedly with cold distilled water until a neutral pH was achieved.

All treated samples were extracted in acidic distilled water (pH of 2.0 by HCl) containing 0.1% *(w*/*v)* pepsin (1:10,000, Wako Pure Chemical Industries Ltd., Osaka, Japan) for 48 h with continuous stirring. A sample/solution ratio of 1:40 (*w*/*v*) was used for the skin and swim bladders, while 1:20 (*w*/*v*) was used for the scales. The extracts were centrifuged at 2000× *g* for 90 min (Model 6800, KUBOTA Manufacturing Corporation, Tokyo, Japan). The resulting precipitates were subjected to repeated extractions under the same conditions until no residue remained in the solutions in the case of the skin and swim bladder samples. The extraction of the scales was stopped after the fourth extraction, though residues still existed. The supernatant was filtered through membrane filters with pore sizes of 3.0, 0.8, and 0.45 µm (Advantec, Tokyo, Japan). Collagen in the filtrate was precipitated by salting it out with NaCl to a final concentration of 1 M. The precipitate was then collected by centrifugation at 2000× *g* for 90 min, and the pellet was dissolved in the acidic distilled water. This purification process was repeated three times to obtain purified collagen. Then, the samples were dialyzed (MWCO 12–14 kDa) against distilled water for 24 h with periodic water changes. The dialyzed collagen suspension was lyophilized using a freeze dryer (FDU-2200; Tokyo Rikakikai Co., Ltd., Tokyo, Japan) and stored at −80 °C until use.

The carp skin, scales, and swim bladders were designated as CSK, CSC, and CSB, respectively. The bester sturgeon skin and swim bladders were designated as SSK and SSB, respectively. The yields, extractability, and purity of each collagen are presented in Supplementary Material S1.

### 4.3. Amino Acid Analysis

The amino acid composition was analyzed at the Instrumental Analysis Division, Equipment Management Center, Creative Research Institution, Hokkaido University. Briefly, the Amino Acid Analyzer L-8900 (Hitachi High-Technologies Corporation, Tokyo, Japan), equipped with a 2620 MPH column (4.6 mm × 80 mm) and an ammonia filter column (2650 L, 4.6 mm × 40 mm), was used for sample analysis. The eluent was a self-made buffer set for standard amino acid analysis with a 0.19 mL/min flow rate. A ninhydrin chromogenic solution (Wako Pure Chemical Industries, Ltd., Osaka, Japan) was used as the reaction reagent at a flow rate of 0.175 mL/min, and the reactions were carried out at 135 °C. Detection was performed at 570 nm (VIS1) for most amino acids and at 440 nm (VIS2) for hydroxyproline and proline. A sample injection volume of 40 µL was used for all analyses. The samples were tested three times, and the means were calculated to determine the amino acid compositions.

### 4.4. Circular Dichroism (CD) Measurements

The CD spectra were measured using a spectrometer (model 725, JASCO, Tokyo, Japan). To determine the triple helical structure of the collagen, freeze-dried collagen was dissolved in acidic distilled water (pH of 3.0, adjusted by HCl) to 0.1 mg/mL. Then, the spectra were measured at wavelengths of 190–250 nm at 10 °C at a scan speed of 50 nm/min, with an interval of 0.1 nm. To determine the denaturation temperature of the collagen molecule, the collagen was dissolved in acidic distilled water (pH 3.0) to 0.5 mg/mL, and the rotatory angle at a fixed wavelength of 221 nm was measured at 10–50 °C at a rate of 0.5 °C/min.

### 4.5. Viscosity Measurements of the Collagen Solutions

The dynamic viscosity of the collagen solution of 4% (*w*/*v*) was assessed using a rheometer (MCR 702e MultiDrive, Anton Paar, Graz, Austria). The measurements were conducted with a plate geometry of a 25 mm diameter and a fixed gap of 0.054 mm. The temperature was controlled and maintained at 20 °C. The shear rate was systematically varied from 0.1 to 10 s^−1^.

### 4.6. Determination of the Gel-Forming Ability

The freeze-dried collagens were mixed with acidic distilled water (pH of 2.5, adjusted by HCl) to 4% (*w*/*v*) and left at 4 °C for 4–5 days to obtain homogeneous solutions. The gel-forming ability of each collagen solution was assessed as shown in Supplementary Material S2. The gel formation was tested at various time intervals (3, 6, 12, and 24 h) at 20 °C.

### 4.7. Scanning Electron Microscopy (SEM)

The surface microstructures of the collagen gels were analyzed using SEM (JSM6010LA, JEOL Ltd., Tokyo, Japan). After gelation, the gels were fixed with 2.5% glutaraldehyde for 3 h at room temperature. They were then rinsed in 0.1 M phosphate buffer (PB) at a pH of 7.2 for 3 h, and the buffer solution was replaced every hour. The samples were subsequently dehydrated in a graded ethanol series (70–100%) for 30 min each, followed by two 30 min rinses in *t*-butyl alcohol. The gels were freeze-dried using a *t*-butyl alcohol solution and a freeze-dryer (FDU-2200, Tokyo Rikakikai Co., Ltd., Tokyo, Japan). Finally, they were coated with gold platinum using an auto-fine coater (JFC-1600, JEOL Ltd., Tokyo, Japan) and examined using SEM.

### 4.8. Mechanical Test of the Gels

The collagen gels with a thickness of 1 mm were prepared (Supplementary Material S2 Figure S3). A dynamic frequency sweep test was conducted using a rheometer (AresG2 TA instruments, New Castle, DE, USA) to assess the gels’ properties. The frequency range was varied from 1 to 100 s^−1^, with a constant shear strain of 0.2%. The measurements were performed using parallel plate geometry at a controlled temperature of 20 °C.

### 4.9. Gel Thermal Stability Analysis

The thermal stability of the gels was evaluated using differential scanning calorimetry (DSC) (EXSTAR DSC6100; SII Nano Technology Inc., Chiba, Japan). Briefly, 0.5 mm thick gels were prepared, and 15–20 mg of each gel sample was weighed and sealed in a 70 µL aluminum pan. Measurements were performed from 25 to 60 °C at a heating rate of 2 °C/min, with a sampling interval of 0.2 s, using phosphate buffer (pH of 7.2) as the reference, under static air conditions without a special gas atmosphere. The denaturation temperature (Td) was defined as the peak maximum of the thermal transition observed in the DSC curve.

## Figures and Tables

**Table 1:** Amino acid compositions of the collagens extracted from carp skin (CSK), scales (CSC), and swim bladders (CSB) and sturgeon skin (SSK) and swim bladders (SSB) (expressed as residues/1000 total amino acid residues).

Amino Acids	CSK	CSC	CSB	SSK	SSB
Aspartic acid	47	48	46	49	48
Threonine	24	26	28	25	27
Serine	36	36	32	45	46
Glutamic acid	68	71	71	69	71
Glycine	338	334	334	346	342
Alanine	121	115	125	115	116
Cysteine	0	1	1	1	1
Valine	19	21	19	17	18
Methionine	13	13	13	10	10
Isoleucine	10	12	11	12	13
Leucine	21	23	21	18	19
Tyrosine	2	3	3	2	2
Phenylalanine	12	13	13	13	13
Hydroxylysine	7	11	8	8	13
Lysine	26	24	26	25	21
Histidine	5	6	4	5	5
Arginine	52	51	52	52	52
Hydroxyproline	79	80	75	63	82
Proline	120	114	118	124	102
Imino acid	199	194	193	187	184

## Data Availability

The data supporting the findings of this study are included in the article and its supplementary files. Further inquiries can be directed to the corresponding author.

## Supplementary Materials

The following supporting information can be downloaded at: https://www.mdpi.com/article/10.3390/gels11070533/s1, Table S1: Yields of collagen purified from carp skin (CSK), scales (CSC), and swim bladders (CSB) and sturgeon skin (SSK) and swim bladders (SSB). Figure S1: Solubilized collagen after each extraction step, expressed as a percentage of the total solubilized collagen obtained. Horizontal strips: first extraction; vertical lines: second extraction; black: third extraction; diagonal bricks: fourth extraction. Figure S2: SDS-PAGE of the collagens from carp skin (CSK), scales (CSC), and swim bladders (CSB) and sturgeon skin (SSK) and swim bladders (SSB). M: high-molecular-weight marker. Figure S3: Experimental setup for diffusion-induced gelation in the fabrication of the collagen gels from carp and sturgeon organs.

## References

[1] Silvipriya K., Kumar K., Bhat A., Kumar B., John A., Lakshmanan P. (2015). Collagen: Animal sources and biomedical application. J. Appl. Pharm. Sci..

[2] Easterbrook C., Maddern G. (2008). Porcine and bovine surgical products jewish, muslim, and hindu perspectives. Arch. Surg..

[3] Salvatore L., Gallo N., Natali M.L., Campa L., Lunetti P., Madaghiele M., Blasi F.S., Corallo A., Capobianco L., Sannino A. (2020). Marine collagen and its derivatives: Versatile and sustainable bio-resources for healthcare. Mater. Sci. Eng. C.

[4] Zhang X., Ookawa M., Tan Y., Ura K., Adachi S., Takagi Y. (2014). Biochemical characterization and assessment of fibril-forming ability of collagens extracted from Bester sturgeon *Huso huso* × *Acipenser ruthenus*. Food Chem..

[5] Dudu A., Georgescu S.E. (2024). Exploring the multifaceted potential of endangered sturgeon: Caviar, meat and by-product benefits. Animals.

[6] FAO (2024). In Brief to the State of World Fisheries and Aquaculture 2024. Blue Transformation in Action.

[7] Peñarubia O., Toppe J., Ahern M., Ward A., Griffin M. (2023). How value addition by utilization of tilapia processing by-products can improve human nutrition and livelihood. Rev. Aquac..

[8] Coppola D., Lauritano C., Palma Esposito F., Riccio G., Rizzo C., De Pascale D. (2021). Fish waste: From problem to valuable resource. Mar. Drugs.

[9] Zhang X., Adachi S., Ura K., Takagi Y. (2019). Properties of collagen extracted from Amur sturgeon *Acipenser schrenckii* and assessment of collagen fibrils in vitro. Int. J. Biol. Macromol..

[10] Matarsim N.N., Jaziri A.A., Shapawi R., Mokhtar R.A.M., Noordin W.N.M., Huda N. (2023). Type I collagen from the skin of barracuda (*Sphyraena* sp.) prepared with different organic acids: Biochemical, microstructural and functional properties. J. Funct. Biomater..

[11] Dong Y., Dai Z. (2022). Physicochemical, structural and antioxidant properties of collagens from the swim bladder of four fish species. Mar. Drugs.

[12] Jaziri A.A., Shapawi R., Mokhtar R.A.M., Noordin W.N.M., Huda N. (2022). Physicochemical and microstructural analyses of pepsin-soluble collagens derived from lizardfish (*Saurida tumbil* Bloch, 1795) skin, bone and scales. Gels.

[13] Ahmed R., Haq M., Chun B.S. (2019). Characterization of marine derived collagen extracted from the by-products of bigeye tuna (*Thunnus obesus*). Int. J. Biol. Macromol..

[14] Pal G.K., Nidheesh T., Suresh P.V. (2015). Comparative study on characteristics and in vitro fibril formation ability of acid and pepsin soluble collagen from the skin of catla (*Catla catla*) and rohu (*Labeo rohita*). Food Res. Int..

[15] Duan R., Zhang J., Du X., Yao X., Konno K. (2009). Properties of collagen from skin, scale and bone of carp (*Cyprinus carpio*). Food Chem..

[16] Zhou C., Li Y., Yu X., Yang H., Ma H., Yagoub A.E.A., Cheng Y., Hu J., Out P.N.Y. (2016). Extraction and characterization of chicken feet soluble collagen. LWT–Food Sci. Technol..

[17] Yu D., Chi C.-F., Wang B., Ding G.-F., Li Z.-R. (2014). Characterization of acid- and pepsin-soluble collagens from spines and skulls of skipjack tuna (*Katsuwonus pelamis*). Chin. J. Nat. Med..

[18] Huang Y.-R., Shiau C.-Y., Chen H.-H., Huang B.-C. (2011). Isolation and characterization of acid and pepsin solubilized collagens from the skin of balloon fish (*Diodon holocanthus*). Food Hydrocoll..

[19] Ikoma T., Kobayashi H., Tanaka J., Walsh D., Mann S. (2003). Physical properties of type I collagen extracted from fish scales of *Pagrus major* and *Oreochromis niloticus*. Int. J. Biol. Macromol..

[20] Menezes M.D.L.L.R., Ribeiro H.L., Abreu F.D.O.M.D.S., Feitosa J.P.D.A., Filho M.D.S.M.D.S. (2020). Optimization of the collagen extraction from Nile tilapia skin (Oreochromis niloticus) and its hydrogel with hyaluronic acid. Colloids Surf. B Biointerfaces.

[21] Shen Z., Zhang Q., Li L., Li D., Takagi Y., Zhang X. (2022). Properties of grass carp (*Ctenopharyngodon idella*) collagen and gel for application in biomaterials. Gels.

[22] Mredha M.T.I., Zhang X., Nonoyama T., Nakajima T., Kurokawa T., Takagi Y., Gong J.P. (2015). Swim bladder collagen forms hydrogel with macroscopic superstructure by diffusion induced fast gelation. J. Mater. Chem. B.

[23] Lai G., Li Y., Li G. (2008). Effect of concentration and temperature on the rheological behavior of collagen solution. Int. J. Biol. Macromol..

[24] Doyle A.D., Yamada K.M. (2016). Mechanosensing via cell-matrix adhesions in 3D microenvironments. Exp. Cell Res..

[25] Zhang L., Li K., Xiao W., Zheng L., Xiao Y., Fan H., Zhang X. (2011). Preparation of collagen–chondroitin sulfate–hyaluronic acid hybrid hydrogel scaffolds and cell compatibility in vitro. Carbohydr. Polym..

[26] Dawlee S., Sugandhi A., Balakrishnan B., Labarre D., Jayakrishnan A. (2005). Oxidized chondroitin sulfate-cross-linked gelatin matrixes: A new class of hydrogels. Biomacromolecules.

[27] Zhang Y., Shen L., Cheng Y., Li G. (2021). Stable and biocompatible fibrillar hydrogels based on the self-crosslinking between collagen and oxidized chondroitin sulfate. Polym. Degrad. Stab..

[28] Liu S., Lau C.-S., Liang K., Wen F., Teoh S.H. (2022). Marine collagen scaffolds in tissue engineering. Curr. Opin. Biotechnol..

[29] Karaoğul E., Ugurtay A., Kelley S.S., Alma M.H. (2025). Modelling on Extractions and Micro-Nano Encapsulation of *Pistacia terebinthus* Cambium Layer Gum Essential Oil: Antioxidant Activity and Structural Approach via XRD, SEM and TGA/DSC. Plant Foods Hum. Nutr..

[30] Meng D., Tanaka H., Kobayashi T., Hatayama H., Zhang X., Ura K., Yunoki S., Takagi Y. (2019). The effect of alkaline pretreatment on the biochemical characteristics and fibril-forming abilities of types I and II collagen extracted from bester sturgeon by-products. Int. J. Biol. Macromol..

