# Supplementary material for: Comparative Study of the Gel-Forming Ability of Type I Collagens Extracted from Different Organs and Fish Species
Source: Gels. 2025 Jul 9;11(7):533. doi: 10.3390/gels11070533 (PMC12294791; doi:10.3390/gels11070533)
Supplement: Supplementary file 1 [file gels-11-00533-s001.zip › Supplementry_material_2.pdf]

Supplementary Material 2

Methods to fabricate carp and sturgeon collagen gels

(A) 20 µL of collagen solution, 4% (w/v) in acidic deionized water (pH 7.2 adjusted by HCl), was placed between two glass coverslips (24 × 50 mm and 24 × 24 mm) separated by 0.5-mm-thick silicone spacers.

(B) Gelation was triggered by introducing 100-mM phosphate buffer at pH 7.2 from the peripheral region of the collagen solution.

(C) The gelation process proceeded from the periphery toward the center through the diffusion of the buffer, resulting in a disk-shaped collagen hydrogel with a diameter of approximately 6 mm and a thickness of 0.5 mm.

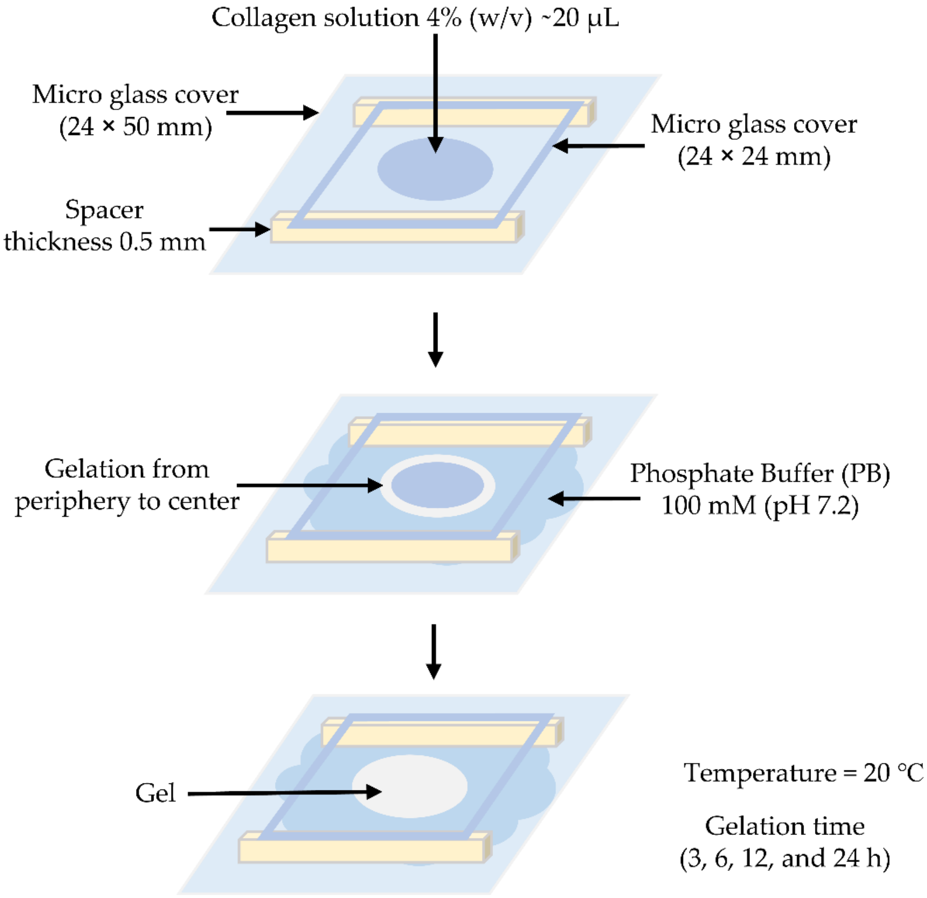

Figure S3. Experimental setup for diffusion-induced gelation in the fabrication of collagen gels from carp and sturgeon organs.
