# Supplementary material for: Comparative Study of the Gel-Forming Ability of Type I Collagens Extracted from Different Organs and Fish Species
Source: Gels. 2025 Jul 9;11(7):533. doi: 10.3390/gels11070533 (PMC12294791; doi:10.3390/gels11070533)
Supplement: Supplementary file 1 [file gels-11-00533-s001.zip › Supplementry_material_1.pdf]

Supplementary Material 1

Yields and extractability of each collagen

Methods

The total collagen yield was determined using the following equation:

$$\text{Yield of collagen (\%)} = \frac{C_t}{W_t} \times 100$$

where C_t is the total extracted collagen and W_t is the initial organ weight (dry weight basis).

To assess the proportion of collagen solubilized in each extraction step, the following equation was used:

$$\text{Solubilized collagen (\%)} = \frac{C_i}{C_t} \times 100$$

where C_i is the collagen extracted in each step, and C_t is the total collagen extracted (on a dry weight basis).

Results

Table S1 presents the yield of each collagen from carp skin (CSK), scale (CSC), swim bladder (CSB), and sturgeon skin (SSK) and swim bladder (SSB). The highest yield was obtained from SSK (31.29%), followed by SSB (24.88%), CSB (17.16%), CSK (14.89%), and CSC (5.75%).

Figure S2 shows the solubilized collagen after each extraction step, expressed as a percentage of the total solubilized collagen obtained. Two extractions were needed for the complete dissolution of CSK, CSB, SSK, and SSB, whereas scale residues were obtained even after the fourth extraction. Thus, we stopped the extraction of CSC collagen. The lower yield of CSC collagen is subjected to the residues obtained even after the fourth extraction.

Table S1. Yields of collagen purified from carp skin (CSK), scale (CSC), swim bladder (CSB), and sturgeon skin (SSK), and swim bladder (SSB).

Samples	Yields (%)
CSK	14.89
CSC	5.75
CSB	17.16
SSK	31.29
SSB	24.88

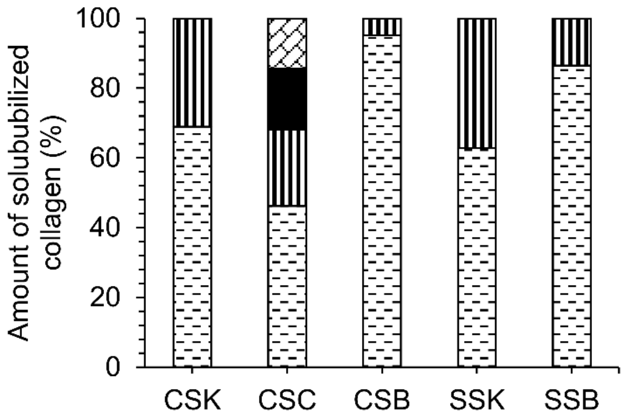

Figure S1. Solubilized collagen after each extraction step, expressed as a percentage of total solubilized collagen obtained. Horizontal strips, first extraction; vertical lines, second extraction; black, third extraction; diagonal bricks, fourth extraction.

Sodium dodecyl sulfate-polyacrylamide gel electrophoresis (SDS-PAGE)

Methods

Lyophilized collagen samples (1 mg/mL) were dissolved in acidic distilled water (pH 3.0 adjusted by HCl) and mixed with buffer (0.5 M Tris-HCl, pH 6.8, containing 4% SDS, 20% glycerol) containing 10% β-mercaptoethanol in a 1:1 ratio (v/v). The mixture was boiled for 3 min, and 5 µg of protein was loaded per lane. Electrophoresis was conducted at 14 mA for the stacking gel and 24 mA for the running gel (7.5%). After staining with Coomassie Brilliant Blue for 30 min, the gel was destained with a solution of 20% ethanol, 5% acetic acid, and 2.5% glycerin. Molecular weight was estimated using Precision Plus Protein All Blue Standard (Bio-Rad Laboratories, Inc., Hercules, CA, USA)

Results

The SDS-PAGE profiles of collagens are presented in Fig. S2. All samples predominantly contained two major bands that correspond to α1 and α2 chains (the α1 band includes the α3 chain). Also, high-molecular-weight bands were observed, likely corresponding to crosslinked α chains in dimers (β chains) and trimers (γ

chains). A comparison of crosslinking patterns among tissues showed that carp tissue collagens had higher crosslinking levels than sturgeon tissues. The CSB collagen displayed the highest degree of crosslinking among the carp tissues, followed by CSK and CSC collagens. Conversely, in sturgeon, the SSB collagen exhibited only trace crosslinking. The results of the current SDS-PAGE analysis suggest that the extent of crosslinking may not fully explain the difficulty in collagen extraction from specific organs. This study also showed that collagen extraction was most challenging from CSC, followed by SSK, CSK, SSB, and CSB (Figure S1).

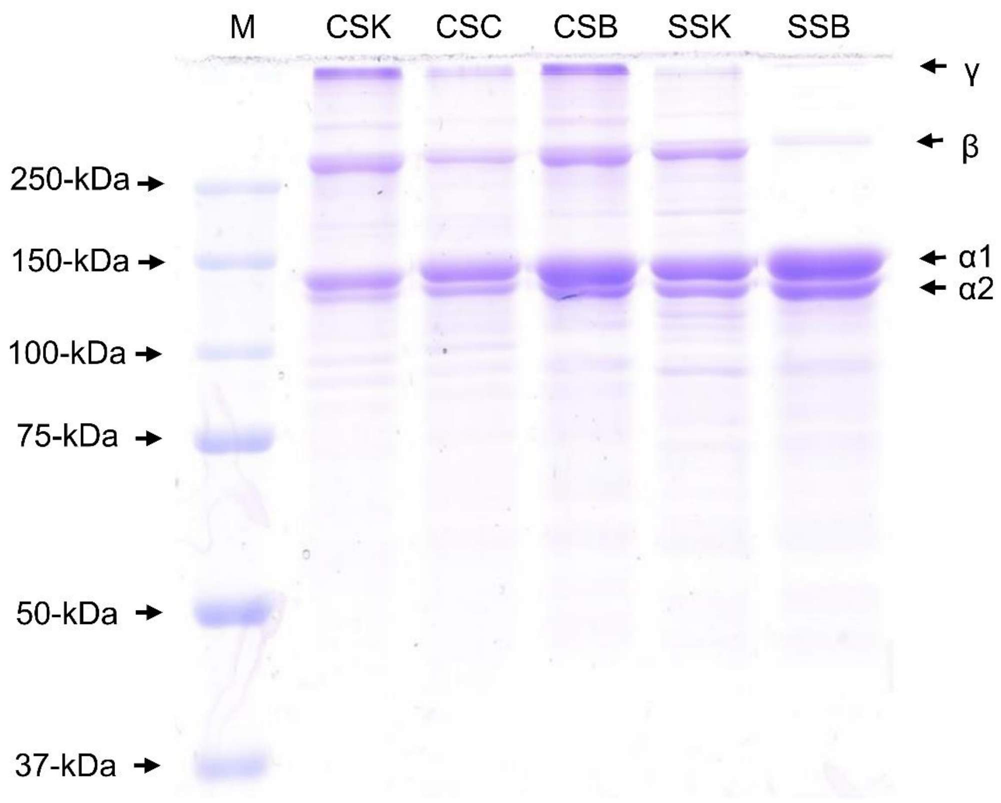

Figure S2. SDS-PAGE of collagens from carp skin (CSK), scale (CSC), swim bladder (CSB), and sturgeon skin (SSK) and swim bladder (SSB). M, high-molecular-weight marker.
